# Personality Traits Moderate the Effect of Workload Sources on Perceived Workload in Flying Column Police Officers

**DOI:** 10.3389/fpsyg.2015.01835

**Published:** 2015-11-27

**Authors:** Carlo Chiorri, Sergio Garbarino, Fabrizio Bracco, Nicola Magnavita

**Affiliations:** ^1^Department of Educational Sciences, University of GenovaGenova, Italy; ^2^State Police Health Service Department, Ministry of the Interior, Department of Neuroscience, Rehabilitation, Ophthalmology, Genetics, Maternal and Child Health, University of GenovaGenova, Italy; ^3^Department of Public Health, Università Cattolica del Sacro CuoreRome, Italy

**Keywords:** personality, workload, first responders, police officers, Five Factor Model

## Abstract

Previous research has suggested that personality traits of the Five Factor Model play a role in worker's response to workload. The aim of this study was to investigate the association of personality traits of first responders with their perceived workload in real-life tasks. A flying column of 269 police officers completed a measure of subjective workload (NASA-Task Load Index) after intervention tasks in a major public event. Officers' scores on a measure of Five Factor Model personality traits were obtained from archival data. Linear Mixed Modeling was used to test the direct and interaction effects of personality traits on workload scores once controlling for background variables, task type and workload source (mental, temporal and physical demand of the task, perceived effort, dissatisfaction for the performance and frustration due to the task). All personality traits except extraversion significantly interacted at least with one workload source. Perceived workload in flying column police officers appears to be the result of their personality characteristics interacting with the workload source. The implications of these results for the development of support measures aimed at reducing the impact of workload in this category of workers are discussed.

## Introduction

Stress occurs when an environmental demand exceeds the natural regulatory capacity of an organism, in particular, situations that include unpredictability and uncontrollability (Koolhaas et al., 2011). Similarly, the European Union defined work-related stress as a worker's experience of not being able to cope with or to control the demand from the work environment (European agency for safety and health at work, 2009).

If prolonged, stress can have adverse psychological (e.g., anxiety, depression) and physical (e.g., headache, gastrointestinal problems) consequences (Nixon et al., 2011; Ganster and Rosen, 2013). Arguably, the two most prominent theoretical models that conceptualized work stress are Karasek's demand-control (or job strain) model (Karasek, 1979) and Siegrist's effort-reward imbalance model (Siegrist, 1996). In both models, a key role is played by workload, which, at least in Europe, is considered one of the most common causes of work-related stress, together with job insecurity (European agency for safety and health at work, 2013). Workload can induce stress because the amount of work to do cannot be accomplished comfortably, because the work is too difficult, or because the work fails to use a worker's skills and abilities (Katz and Kahn, 1978). There is not a generally agreed definition of workload, since it is a multifaceted construct that could be tackled focusing on at least one of three main factors: (i) task demands/task difficulty; (ii) operator workload and strain; (iii) task performance (Megaw, 2005). In this paper we focus on a human-centered definition of mental workload, which assumes that workload is not an inherent property of the task, but rather it emerges from the interaction between the requirements of a task, and thus summarizes the influences of many factors in addition to the objective demands imposed by the task (Hancock and Chignell, 1986). According to Hart and Staveland (1988), the operator could be affected by the perceived task demands after a cognitive appraisal of the task nature, the environment where it is taking place, social, and organizational factors that could interact as stressors. The amount of perceived mental stress depends on operators' characteristics like attitudes, motivation, achievement level, skills, knowledge, expertise, and physical conditions (Megaw, 2005). The interaction between task demands and operator's characteristics causes a mental strain, affecting the performance in terms of accuracy, reliability and efficiency. The evaluation of the performance outcomes, in turn, can influence the perceived workload in terms of frustration and reallocation of resources.

Every assessment of mental workload should take into account the multifaceted nature of the construct. Hart and Staveland (1988) pointed out that workload represents a collection of sources (or attributes) that may be relevant for the explanation of performance, but they should be investigated in their mutual relationship. Accordingly, the experienced workload could be a combination of the following sources:

1. Task-related characteristics:
  - *Mental demand*: the level of cognitive processing required by the task (e.g., reasoning, calculating, remembering, searching, etc.);
  - *Physical demand*: the physical activity needed to accomplish the task (e.g., pushing, running, lifting weights, etc.);
  - *Temporal demand*: the time pressure experienced during the task (e.g., the rate or pace of the events);
2. Behavior-related characteristics (*Performance*): the subjective assessment of success in achieving the goals of the task, the satisfaction in accomplishing them;
3. Operator-related characteristics: the psychological impact on the operator of the task demands, causing:
  - *Frustration*: the feeling of being insecure, discouraged, stressed and annoyed by the task;
  - *Effort*: the subjective feeling of the effort the operator had to provide in order to accomplish the desired level of performance.

In Karasek's model workload is included as the demand component, while in Siegrist's model as the effort component, i.e., the obligations the worker is faced with. In either case, workload acts as a stressor, whose effect can be moderated by other work characteristics as control or reward, respectively. However, perceived workload can be influenced also by individual differences in stress reactivity and stress responses (Lazarus and Folkman, 1984). As stress arises from the interplay between individual and task characteristics (Scheier and Carver, 1987), some workers might be more vulnerable to stress than others. Individual characteristics are involved in all stages of the stress process and a number of models have been proposed to account for the different role they may play (see Section 1 of the Supplementary Material for a more detailed review of these studies). As a result, recent studies provided evidence of the importance and the need to include individual differences in the Karasek's (Schaufeli and Taris, 2014) and Siegrist's (Allisey et al., 2012; Törnroos et al., 2012, 2013) models.

Occupational research has often focused on the role played by personality traits i.e., habitual patterns of behaving, thinking, and experiencing emotions that tend to be relatively stable over time, differ across individuals and influence behavior (Matthews et al., 2003) in moderating the stress response. In the last decades the so-called Five Factor Model (FFM) of personality has emerged and has been accepted as a wide conceptual framework for integrating all the research findings and theories in personality psychology. It assumes that individual differences in adult personality characteristics can be organized in terms of five broad trait domains: Extraversion (i.e., being sociable, gregarious, assertive, talkative, and dynamic), Agreeableness (i.e., being courteous, flexible, trusting, good-natured, cooperative, forgiving, and tolerant), Conscientiousness (i.e., being dependable, hard-working, achievement-oriented, and persevering), Neuroticism (i.e., being anxious, depressed, emotionally unstable, worried, and insecure) and Openness to experience (i.e., being imaginative, cultured, curious, original, broad-minded, intelligent, and artistically sensitive).

A huge body of empirical research has supported the stability and predictive validity of the FFM traits across different populations, settings, and countries (e.g., McCrae and Costa, 1997). Moreover, it has been shown that these traits are invariant across age and gender, and have a biological-heritable basis (Costa and McCrae, 1992). In organizational psychology, this suggested a link to the physiological process underlying stress-related illness and disease, and it has been hypothesized that FFM traits play an important role in workers' exposure to workload, stress, cognitive appraisal, coping, health, and well-being (Grant and Langan-Fox, 2007). While the results about four out of five personality traits were not consistently replicated, Neuroticism has emerged as a key trait, since individuals scoring high on this trait (high Ns) tend to respond poorly to environmental stress, to perceive ordinary situations as threatening, and to experience even minor frustrations as hopelessly overwhelming (e.g., Widiger, 2009).

Eysenck and Eysenck (1985) suggested that high Ns often show a discrepancy between performance efficiency (i.e., the amount of processing resources invested in a task) and performance effectiveness (i.e., the quality of the performance). High Ns can exhibit the same level of performance effectiveness as low Ns, but they need a higher resource and effort expenditure, suggesting that resource investment may result from increasing motivational incentives or individual differences, such as attitudes and personality traits (Vidulich and Wickens, 1986). More recently, Rose et al. (2002) reported that, while Extraversion and Conscientiousness were associated with performance measures, Neuroticism positively correlated with frustration in a vigilance performance task. Szalma and Taylor (2011) also found that Neuroticism generally predicted increased workload and stress in adaptive automation tasks. Szalma (2009) found that pessimism (which can be considered as a proxy for Neuroticism) influenced overall workload in a focused task, and marginally in an integration task, such that higher levels of pessimism were associated with higher overall workload. More specifically, in the focused task pessimism was related to higher dissatisfaction for the performance and higher frustration. This was consistent with previous findings that pessimists showed a tendency to rate their own performance as poor and tended to report more negative affect (Szalma, 2009).

As for the other traits, Grant and Langan-Fox (2007) found that higher Extraversion was associated with higher physical health and higher job satisfaction, whereas Neuroticism was associated with lower physical health and lower job satisfaction. Contrary to predictions, Conscientiousness did not directly contribute to the prediction of physical ill health and job satisfaction over and above Extraversion and Neuroticism. Agreeableness was unrelated to physical ill health and did not contribute uniquely to the prediction of job satisfaction after controlling for Extraversion, Neuroticism, and Conscientiousness. Openness was unrelated to physical ill health or job satisfaction, with the findings for job satisfaction supporting past research. Conard and Matthews (2008) found that Neuroticism was the strongest predictor of stress, regardless of whether the stress was cognitive or emotional, and regardless of the level of stressors present. Similar results were obtained by van Emmerik (2008), who reported that Neuroticism and Openness to experience were positively related to time-related strains. More recently, Szalma and Teo (2012) found that Extraversion moderated the relationship of task characteristics to performance, global workload, distress, and task engagement in a cognitive signal detection task. Specifically, an increasing task load resulted in a performance benefit for participants who are higher in Extraversion. According to the authors, this can be considered as evidence that tasks, by themselves, do not determine stress response but, rather, that the characteristics of the individual interact with task parameters to determine the response to stress.

While there is a wide body of research on the role played by personality traits on stress response in many different occupations, little seems to be known about high-risk occupations, i.e., those occupations that present volatility, uncertainty, complexity and ambiguity of general conditions and situations (the so-called VUCA) and the possibility of substantial and unpredictable danger. This category includes first responders, peacekeepers, and emergency workers such as police officers, soldiers, fire-fighters, emergency medical staff, etc., whose main tasks are to protect human life, property and the environment in everyday emergencies, natural disasters, industrial and transport accidents, terrorist and criminal attacks, and massive public events. Although reliable statistics are not available, the increasing number of people affected by small scale emergencies and major disasters indicate that a significant proportion of the workforce is involved in emergency activities and disaster control all around the world (Milczarek, 2011).

Unlike other kinds of workers, these workers face some specific occupational safety and health hazards. Their work environment is very demanding, due to the high probability of working in remote and dangerous areas and the high risk of violence. As a result, they are exposed to severe emotional, psychological and physical overstrain, due to threats to their health and safety, responsibility for protecting the lives of others, sudden shifts from boredom to alertness and mobilized energy, exposure to people in pain or distress (Milczarek, 2011). Previous studies on these populations mainly focused on the long term health consequences of this kind of work (for a review, see Benedek et al., 2007). For instance, Peng et al. (2012) tested a model that linked personality to coping and distress on a sample of military personnel deployed in Iraq during a peak period in the fighting. They found that Conscientiousness, Neuroticism, and Extraversion were associated with different coping behaviors, and these were in turn related to psychological distress.

However, there seem to be a paucity of studies that investigated the role of personality traits in the perception of workload, although it has been acknowledged as a crucial factor in achieving and maintaining performance success in first responders (Boermans et al., 2013). Among the few exceptions, Kitamura et al. (2013) reported that together with substantial workload, individual differences in emotional stability (i.e., the opposite pole of Neuroticism) and in resilience had an impact on perceived fatigue of local government employees who had responded to two natural disasters. The authors argued that these individual factors should be considered as potential moderators of distress among local government employees responding to disasters. This is consistent with Beaty et al.'s (2001) claim that the role of personality appears to be crucial when environments are less clearly structured in terms of prescribed behavior, and thus individual personality characteristics are relied on to direct behavior. Other studies investigated the effects of personality traits when dealing with high demands at the workplace using diary data. However, they almost always considered only Neuroticism and/or Extraversion, and investigated other kinds of workers. For instance, Rubino et al. (2012) found that individuals high in Neuroticism did either not benefit as readily from decision latitude^1^ or were more susceptible to job demands when they had decision latitude. Zhou et al. (2015) reported that daily workplace incivility positively predicted end-of-work negative affect while controlling for before-work negative affect, and that this effect was stronger in individuals with high Neuroticism, hostile attribution bias, and external locus of control.

The aim of the present study was to test the effect of *all* FFM traits on perceived workload in activities during a high-risk event of a flying column^2^ of Italian police officers (see Section 2 of the Supplementary Materials for details). This offered a unique opportunity to investigate how basic psychological characteristics contribute to the subjective experience of workload of a group of first responders, once controlling for background variables, task type, and workload source (WS).

We hypothesized that personality traits could have both a direct and a moderator effect. A direct effect would be supported by finding a significant main effect of a trait, i.e., each trait has an additive role in the model as it contributes its own unique variance. However, personality traits could also play a moderator role, i.e., they might affect the direction and/or the strength of the (expected) association between the factors (in this case, task type, and WS) and perceived workload. This hypothesis would be supported by a significant interaction effect of each personality trait with either of the factors. Although this study had a mainly exploratory purpose, since there are no previous studies that addressed the same issues in flying column police officers (FCPOs) in actual duty, grounding on previous studies we could expect that:

- High levels of Extraversion should be associated with high levels of energy and dynamism, with more effective and active coping, with acceptance of responsibility (DeLongis and Holtzman, 2005), with more positive job-related affect, with a more positive appraisal of situations, with a higher satisfaction for performance (Grant and Langan-Fox, 2007). Hence, we expected that officers higher in Extraversion would experience less fatigue and perceive tasks as less stressful and with less time pressure (Wayne et al., 2004). However, we could also expect Extraversion to play a moderator role, since it has been found that for individuals higher in this trait an increasing task load resulted in a performance benefit, whereas unstimulating tasks were perceived as aversive (Szalma and Teo, 2012);
- Officers high in Agreeableness should be better in coping with interpersonal stressors (DeLongis and Holtzman, 2005) and in gaining social support (Barrick and Mount, 1991), which should consequently reduce workload. Hence, it could be predicted that higher Agreeableness would be associated with lower perceived workload, at least in those tasks that may include confrontation with others;
- Officers high in Conscientiousness should show a more careful planning, a more effective organization, a more efficient time management, a higher problem-focused coping ability (Grant and Langan-Fox, 2007). Moreover, as individuals high in Conscientiousness tend to create structure for themselves, they are also less vulnerable to situational role ambiguity (Miller et al., 1999). These characteristics should facilitate positive experience in the work domain and this should reduce the perceived workload, at least in terms of temporal demands (Wayne et al., 2004). However, a high Conscientiousness might exacerbate the effect of underutilization an/or poor team performance (Grant and Langan-Fox, 2007). Given that those who score high on this trait are rigorous, ambitious, goal-oriented, and with self-imposed high standards of performance, they are likely to be frustrated by not achieving their expected level of performance;
- Officers high in Emotional Stability should experience more positive emotions, less negative strain reactions (Cano-Garcia et al., 2005), higher levels of problem solving, lower levels of confrontation, escape avoidance, and self-blame (O'Brien and DeLongis, 1996), more efficient time use, lower preoccupation with role demands, and decreased perceptions of or experience of stress (Wayne et al., 2004). Moreover, this trait has been found to be negatively related to threat appraisal and positively related to challenge appraisal (Gallagher, 1990) and to perceived coping ability (Penley and Tomaka, 2002). Hence, more emotionally stable officers should experience less workload, less dissatisfaction for the performance, less stress, less frustration, and less effort (Rose et al., 2002; Szalma, 2002; Grant and Langan-Fox, 2007; Conard and Matthews, 2008; van Emmerik, 2008). Previous results are not consistent about whether the effect of Emotional Stability is direct, hence suggesting that it could buffer the effect of stress on strain regardless of stressor type (Grant and Langan-Fox, 2007). This trait may also play a moderator effect as in Parkes (1990), where more emotionally stable individuals perceived their work environment as being generally less stressful, and also showed a less reactive response to the same level of perceived demands with respect to less emotionally stable individuals;
- Previous research showed that Openness to experience was positively related to time pressure (van Emmerik, 2008), as individuals high in Openness are more likely to engage in too many activities, making them vulnerable to workload due to time constraints. This might lead to expect that higher Openness would be associated to higher workload. However, Penley and Tomaka (2002) found that Openness was negatively related to perceived situational demands (primary cognitive appraisal) and positively related to perceived coping ability (secondary cognitive appraisal). Given the paucity of effects of Openness reported in the organizational literature, we refrain from providing specific hypotheses about this trait.

## Materials and methods

### Participants

Participants were 269 male^3^ officers of an Italian police flying column, the “VI Reparto Mobile” (VI-RM) of Genoa, a middle-sized city in Northwestern Italy, that were employed in riot control tasks during a high-risk event. Descriptive statistics of the background characteristics of these participants are provided in Section 4 of the Supplementary Materials.

### Personality measure

Scores on personality traits were obtained from the Big Five Questionnaire (BFQ, Caprara et al., 1993). These scores are contained in the database described in Section 2 of the Supplementary Materials and were collected in January 2009. The BFQ is an Italian measure of the FFM traits. It consists of 132 short statements organized in five “domain” scales (24 items each) and one validity scale (12 items). Half of the items are negatively worded. In the BFQ the Big Five are labeled Energy, Friendliness, Conscientiousness, Emotional Stability, and Openness. Each domain contains two facets, but they were not considered in this study since the focus was on the effect of the domains. The domain scale Energy refers to the factor usually labeled as Extraversion, and high scores in this domain are associated with high activity, sociability, enthusiasm, assertiveness, and self-confidence. The domain scale Friendliness refers to the factor usually labeled as Agreeableness and high scores in this domain are associated with high concern and sensitiveness toward others' needs and with high kindness and trust. The domain scale Conscientiousness refers to self-regulation in both its proactive and inhibitory aspects, and high scores in this domain are associated with high dependability, precision, and capability of fulfilling one's own tasks and commitments. The domain scale Emotional Stability refers to personality characteristics often reverse keyed as Neuroticism and high scores in this domain are associated with high capability to cope adequately with one's own anxiety and emotionality and to control irritation and anger. The domain scale Openness refers to the factor labeled as Culture or Intellect or Openness to experience, and high scores in this domain are associated with high broadness of one's own cultural interests, high openness to novelty and high interest toward different people, habits and lifestyles. Participants are asked to rate the degree to which each item adequately describes them on a 5-point Likert-type scale ranging from complete disagreement (1 = very false for me) to complete agreement (5 = very true for me). The BFQ has shown adequate convergent and discriminat validity as Caprara et al. (1993) found high (*r* > 0.60) correlations with homologous dimensions of the NEO-PI-R (Costa and McCrae, 1992) and the Eysenck Personality Inventory (Eysenck and Eysenck, 1975), and absence of high correlations with dimensions tapping different aspects of personality. In this study internal consistencies (Cronbach's as) were: Energy = 0.71, Friendliness = 0.81, Conscientiousness = 0.82, Emotional Stability = 0.87, Openness = 0.79. Consistent with the Caprara et al. (1993)'s study, factor correlations ranged from 0.10 to 0.35.

### Tasks

Tasks were actual activities carried out by the officers in preparation to and in the maintaining of public order during a high-risk, major public event. While Redmans were performed as training drills in the week before the event (July 1st–July 7th, 2009), the other three tasks were performed during the event between July 8th and 10th, 2009.

#### Redman (RED)

Redman is a planned activity consisting in a series of flying-column-specific training programs of varying difficulty. This task was designed to simulate realistic high-risk scenarios and prepare officers for critical decision-making when real-life confrontations occur during, e.g., public demonstrations and rallies. The training lasted approximately 6 h and consisted in performing individual (e.g., using weapons, fighting) or group (tactical strategies for riot control) tasks that were both psychologically and physically demanding. A debriefing was carried out at the end of the training to review and discuss errors and problems.

#### Rapid response (RR)

The officer stayed at the police station and was on 24-h operational duty wearing full riot equipment during a high risk event (e.g., demonstration), ready to be called to intervene.

#### Operational service without intervention (OSw/o)

This was a planned routine activity during usual working hours with rest hours. Tasks typically involved patrolling potentially high-risk targets such as city or region council halls, embassies, monuments, etc.

#### Operational service with intervention (OSw)

This was an emergency intervention that required active control and containment of protesters and could involve physical confrontation, with a high risk of being wounded or injured.

### Subjective workload measure

Hart and Staveland (1988)'s NASA Task Load Index (NASA-TLX, Italian version in Bracco and Chiorri, 2006) is a multi-dimensional rating procedure that provides a subjective measure of mental workload. Since workload may be caused by many different sources (see Introduction), the NASA-TLX requires participants to evaluate them individually: Mental Demand (amount of mental and perceptual activity required to perform the task), Temporal Demand (the amount of time pressure due to the pace at which the tasks or task elements occurred), Physical Demand (the amount of physical activity required), Effort (how hard the worker had to work to accomplish her/his level of performance), Performance (the level of personal satisfaction/dissatisfaction with the performance), and Frustration (the extent to which the person felt irritated, stressed, or annoyed). After performing a task, the participants are presented with six 20-point rating scales (one for each WS) with descriptions of the WSs and two endpoint descriptors, from “low” on the left to “high” on the right (for Performance: from “good” to “poor”). They are asked to evaluate the task by marking each scale at the point that matches their perceived contribution of that WS to their workload. In order to refine the measurement and resolve tied ratings, participants are also presented with all possible pairs (15) of rating scale titles (for example, Effort vs. Mental Demands) and asked to choose which of the two WSs was the more important contributor to their experience of workload in the task they have just performed. The results of these paired comparisons are used to compute weights for each WS by tallying the number of times each WS has been selected and dividing this frequency by 15. The raw rating score is then multiplied by 5 and by the weight to obtain the weighted score. Hence, the highest possible score for a WS is 33.333 (rated as 20 and chosen as the most important contributor to workload in all its five paired comparisons). A total NASA-TLX score can also be computed by adding the weighted WS scores, but this score was not considered in this work, since the internal consistency of the total NASA-TLX scores was very low in three of the tasks (*RR* = 0.60; *OSw/o* = 0.29; *OSw* = 0.24; *RED* = 0.03).

### Procedure

Officers were asked to complete a NASA-TLX soon after each task in the days of the event. The order of tasks could not be randomized, nor the time of measurement could have been kept constant for all officers, since operational needs obviously came first. Moreover, some officers completed the same task more than once, whereas some others never completed some of the tasks. In total we collected 7842 data points (single NASA-TLX scale scores) from 1307 NASA-TLX score sets (which correspond to the total number of tasks performed by the 269 officers). Officers were then asked to provide their matriculation number for matching their NASA-TLX scores with their background information and BFQ scores from the unrestricted database. All of them accepted and gave written informed consent after being presented with a detailed description of the procedure, of the aims of the study and of how the confidentiality of their records and personal information would be managed. This study was carried out in accordance with the Ethical Principles of Psychologists and Code of Conduct (American Psychological Association, 2010) and the Declaration of Helsinki (World Medical Association, 1964/2013). The Ethics Committee of the Università Cattolica del Sacro Cuore of Roma (Italy) approved the study design.

### Statistical analyses

**Figure 1:**
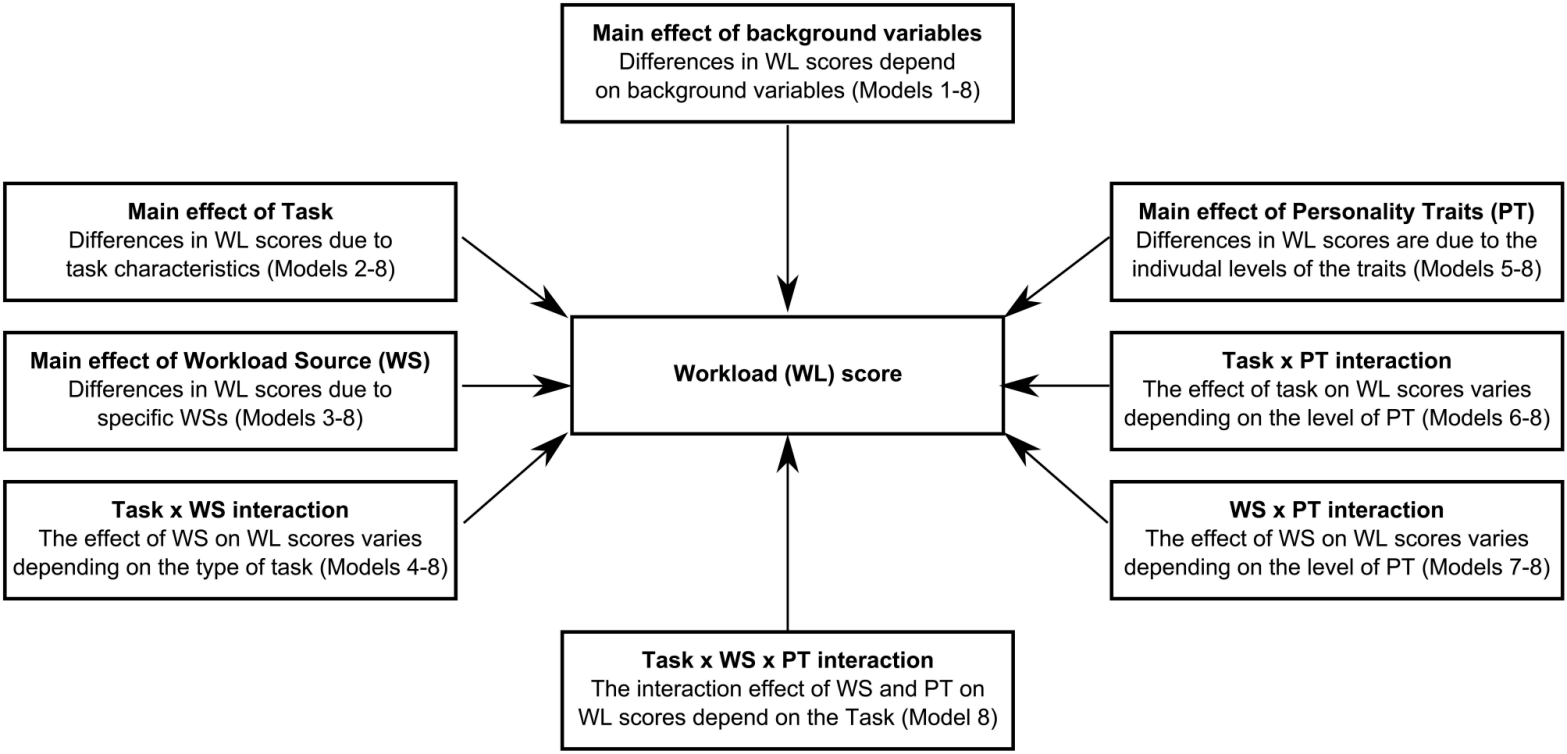
**Diagram with all the effects specified in the Linear Mixed Models used to test the effects of task, workload source, personality variables, and their interactions on perceived workload scores while controlling for background variables**. For a description of the models, see text (Section Statistical Analyses).

The aim of the statistical analyses was to test the effects of task, WS, personality variables, and their interactions on perceived workload scores while controlling for background variables (Figure 1).

This aim can usually be accomplished using a general linear model (e.g., an analysis of covariance or a multiple regression model), but in this case we had to take into account that some officers completed the same task more than once, whereas some others never completed some of the tasks. Hence, we allowed a multilevel structure to data, in which WS scores in each task (Level 1) were nested into officers (Level 2) and used Linear Mixed Modeling (LMM). LMM does not require participants to be measured on the same number of conditions and addresses the issue of non-independence of participants' scores at each measurement occasion through the variance/covariance structure of the model (Gibbons et al., 2010). We considered as dependent variable the level of workload caused by each workload source (WS). Given the scaling procedure of the NASA-TLX, the scores in the different scales are commensurable, and this allows testing a main effect of WS in which the null hypothesis assumes the workload score on all the WS is the same, independently of the other predictors, i.e., if each WS causes the same level of perceived workload. If the interaction effect of WS by a FFM factor is not significant, it means that the effect (slope) of that FFM factor on perceived workload score is the same regardless of the WS.

Note that we report here only the results obtained from random intercepts models. In this study a random intercepts model is a model in which intercepts are allowed to vary across participants, i.e., perceived workload scores for each individual WS in each task are predicted by the intercept (i.e., the mean level of workload perceived by the participant, regardless of the predictors) that varies across participants. We also tested models with both random intercepts and random slopes (i.e., the slope of the effects of the predictors varied across participants, together with the intercept). These models assume that the effects of the predictors vary between clustering units (in our case, the officers), but the fit of the random-intercepts-and-slopes model did not improve enough with respect to a random-intercept-only model to suggest substantial variations of the effects across participants. Hence, these results are not reported.

In order to test how much each independent variable added to the prediction of perceived workload scores we adopted a hierarchical strategy, i.e., a sequential process involving the entry of predictor variables into the model in steps. At each step the researcher can choose which predictors are to enter the model grounding on theory and/or past research (Cohen et al., 2003).

In the first step (Model 1) we entered all the background variables (Age, Educational level [medium, high], Marital status [single, married, divorced], Having children [yes, no], Residence in the region were the unit was based [yes, no], Role [agent, head, technician], Years in service, Being quartered in barracks [yes, no]). In the second step (Model 2) we entered the main effect of task, as this was an exogenous variable (i.e., a variable on which officers had no control). In the third step (Model 3) we entered the main effect of WS. In the fourth step (Model 4) we entered the task by WS interaction effect. This model allowed us to test whether the effect of task varied as a function of the WS considered. In the fifth step (Model 5) we entered the main effects of each personality trait. This model allowed us to test direct effects, i.e., whether each personality trait could independently and significantly predict perceived workload over and above the effects of task and WS. In the sixth step (Model 6) we entered the task by personality trait interaction effects. This model allowed us to test whether personality traits moderated the effect of task on perceived workload. In the seventh step (Model 7) we entered the WS by personality trait interaction effects. This model allowed us to test whether personality traits moderated the effect of WS on perceived workload. In the eighth and final step (Model 8) we entered the task by WS by personality trait interaction effects. This model allowed us to test whether the level of a personality trait could moderate the interaction effect of task and WS.

At each step we evaluated the statistical significance of each predictor and its parameters and the difference in model fit with the previous step. As models were nested within each other, i.e., the parameters of the model at step k were a subset of the parameters of the model at step k+1, we could perform model comparison using the difference in deviance, i.e., a measure of the lack of fit between the data and the model which is obtained by multiplying the natural log of the likelihood (LL, i.e., what is minimized in Maximum Likelihood estimation of multilevel models) by minus two. The difference of the deviances from each model is distributed as a chi-square statistic with degrees of freedom equal to the difference in the number of parameters estimated in each model. Since deviance systematically decreases as the number of parameters in the model increases, in order to provide a more detailed assessment of the parsimony of the models we inspected also the Akaike Information Criterion (AIC) and the Schwarz's Bayesian Information Criterion (BIC), which are based on the deviance but incorporate a penalty for a greater number of parameters. Lower AIC and BIC values indicated a better model (Luke, 2004).

Statistical analyses were carried out with the R packages lme4 (Bates et al., 2014), lmerTest (Kuznetsova et al., 2014) and LMERConvenienceFunctions (Tremblay and Ransijn, 2014).

## Results

**Figure 2:**
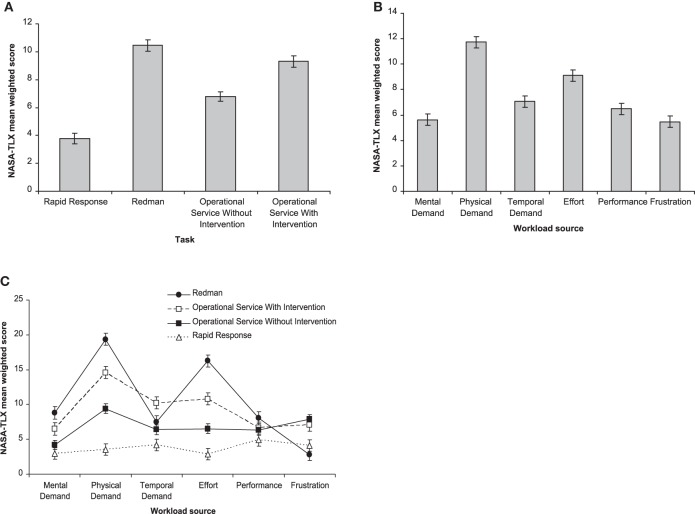
**Estimated marginal means from Model 7**. **(A)** main effect of task; **(B)** main effect of workload source; **(C)** Workload source by task interaction plot.

None of the background variables was significantly associated with perceived workload (the complete set of results is reported in Section 5 of the Supplementary Materials). Hence, Model 1 did not show a significantly better fit than a random intercept-only model [*X*${}_{\left(11\right)}^{2}=15$.93, *p* = 0.143]. When the main effect of task was entered in the model (Model 2), there was a significant increase in fit [*X*${}_{\left(3\right)}^{2}=733$.14, *p* < 0.001], as the main effect of task was statistically significant. *Post-hoc* tests showed that perceived workload was significantly different between any pair of tasks and that RED and OSw were the tasks with the highest perceived workload (Figure 2A).

The entry of the main effect of WS in Model 3 also yielded a significant increase in model fit [*X*${}_{\left(5\right)}^{2}$ = 568.81, p < 0.001], as the main effect of WS was statistically significant. Post-hoc tests showed that perceived workload was significantly different between any pair of WS except Mental Demands vs. Performance and Physical Demand vs. Performance. Temporal Demands and Effort were the WS that most contributed to perceived workload (Figure 2B).

In Model 4 the interaction effect of task with WS was also specified and it yielded a further significant increase in model fit [*X*${}_{\left(15\right)}^{2}=866$.13, *p* < 0.001], as it was statistically significant. *Post-hoc* tests showed that RED was the task that induced the highest levels of perceived Physical Demand and Effort, followed by OSw. OSw/o, and OSw were perceived as more frustrating than RED and RR. The perceived workload of tasks did not substantially differ in Mental Demand, Temporal Demand, and Performance (Figure 2C).

In Model 5 personality scores were entered in the model. The model fit significantly increased [*X*${}_{\left(5\right)}^{2}=16$.47, *p* = 0.006], but only three out of five traits were significant predictors of perceived workload: Extraversion, Conscientiousness and Emotional Stability. Regardless of task and WS, higher levels of Extraversion were associated with higher levels of perceived workload, whereas higher levels of Conscientiousness and Emotional Stability were associated with lower levels of perceived workload.

In Model 6 we entered in the model the interaction effects of personality traits with task to test whether personality moderated the effect of task. This hypothesis was not supported as the model fit did not significantly increase [*X*^2^_(15)_ = 11.48, *p* = 0.718], and none of the parameters was statistically significant. ES was the only main effect of a personality trait that was still statistically significant.

**Figure 3:**
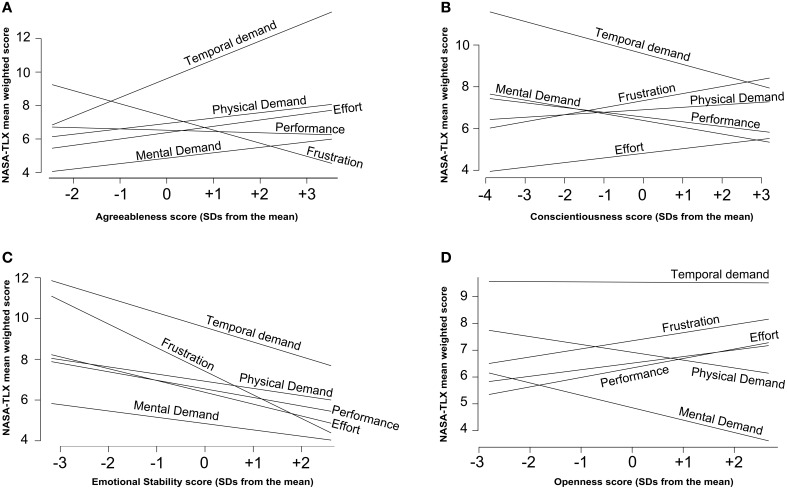
**Interaction effects of personality traits with workload sources**. **(A)** Agreeableness; **(B)** Conscientiousness; **(C)** Emotional Stability; **(D)** Openness.

However, when in Model 7 we entered in the model the interaction of personality traits with WS there was a significant increase in model fit [*X*^2^_(25)_ = 73.06, *p* < 0.001], supporting the hypothesis that personality moderates the effect of WSs. The inspection of parameters showed that there was no significant interaction effect of extraversion with WS, but also that: (a) higher levels of Agreeableness were associated with higher levels of perceived temporal demand and lower levels of frustration (Figure 3A); (b) higher levels of Conscientiousness were associated with lower levels of perceived temporal demand (Figure 3B); (c) higher levels of Emotional Stability were associated with lower levels of perceived frustration (Figure 3C); (d) higher levels of Openness were associated with higher levels of perceived effort, dissatisfaction for the performance and frustration and lower levels of Mental Demand (Figure 3D). No main effect of personality trait was statistically significant (Table 1).

**Table 1:** **Results of hierarchical linear mixed-effects models (Model 7) for predicting weighted workload ratings from background variables, task type, workload source, personality trait scores, and their interactions**.

**Predictor**	**Omnibus effect**	**B**	**SE**
Age		0.04	0.03
Marital Status	*F*_(2, 269.8)_ = 0.95		
Married		0.00	0.35
Divorced		0.63	0.51
Having children		0.04	0.36
Residence in the region		−0.14	0.26
Education	*F*_(2, 254.5)_ = 0.12		
Medium		−0.09	0.34
High		0.13	0.65
Role	*F*_(2, 281.8)_ = 1.78		
Heads		−0.79.	0.47
Technician		0.03	0.57
Years in service		−0.01	0.03
Quartered in barracks		0.12	0.29
Task	*F*_(3, 7787.4)_ = 305.97^***^		
RED		3.98^***^	0.51
OSw/o		−1.46^**^	0.49
Osw		1.13^*^	0.52
WS	*F*_(5, 7572.9)_ = 155.72^***^		
TEM		4.82^***^	0.43
PHY		2.08^***^	0.43
EFF		1.55^***^	0.43
PER		1.69^***^	0.43
FRU		2.47^***^	0.43
Task × WS	*F*_(15, 7572.9)_ = 61.36^***^		
RED × TEM		6.01^***^	0.72
OSw/o × TEM		−4.77^***^	0.70
OSw × TEM		3.90^***^	0.73
RED × PHY		−3.34^***^	0.72
OSw/o × PHY		−1.18.	0.70
OSw × PHY		2.03^**^	0.73
RED × EFF		5.41^***^	0.72
OSw/o × EFF		−2.23^**^	0.70
OSw × EFF		3.16^***^	0.73
RED × PER		−2.25^**^	0.72
OSw/o × PER		0.26	0.70
OSw × PER		−1.71^*^	0.73
RED × FRU		−8.17^***^	0.72
OSw/o × FRU		−1.36.	0.70
OSw × FRU		−1.19	0.73
**PERSONALITY TRAITS**			
EXT		0.12	0.28
AGR		0.32	0.31
CON		0.22	0.27
ES		−0.31	0.28
OPE		−0.46	0.30
Task × EXT	*F*_(3, 7767.4)_ = 0.49		
RED × EXT		−0.12	0.26
OSw/o × EXT		0.21	0.26
OSw × EXT		0.12	0.26
Task × AGR	*F*_(3, 7755.3)_ = 0.07		
RED × AGR		−0.04	0.29
OSw/o × AGR		0.08	0.28
OSw × AGR		−0.05	0.30
Task × CON	*F*_(3, 7777.2)_ = 1.45		
RED × CON		−0.22	0.25
OSw/o × CON		−0.50	0.24
OSw × CON		−0.29	0.25
Task × ES	*F*_(3, 7797.1)_ = 1.56		
RED × ES		0.37	0.26
OSw/o × ES		0.37	0.25
OSw × ES		0.51	0.27
Task × OPE	*F*_(3, 7765.8)_ = 0.66		
RED × OPE		−0.05	0.29
OSw/o × OPE		−0.30	0.28
OSw × OPE		−0.32	0.29
WS × EXT	*F*_(5, 7572.9)_ = 0.48		
TEM × EXT		0.11	0.33
PHY × EXT		−0.02	0.33
EFF × EXT		−0.02	0.33
PER × EXT		0.04	0.33
FRU × EXT		0.40	0.33
WS × AGR	*F*_(5, 7572.9)_ = 5.88^***^		
TEM × AGR		0.81^*^	0.37
PHY × AGR		0.00	0.37
EFF × AGR		0.06	0.37
PER × AGR		−0.40	0.37
FRU × AGR		−1.11^**^	0.37
WS × CON	*F*_(5, 7572.9)_ = 2.34^*^		
TEM × CON		−0.74^*^	0.31
PHY × CON		−0.10	0.31
EFF × CON		−0.55	0.31
PER × CON		−0.45	0.31
FRU × CON		0.12	0.31
WS × ES	*F*_(5, 7572.9)_ = 2.44^*^		
TEM × ES		−0.41	0.33
PHY × ES		−0.04	0.33
EFF × ES		−0.27	0.33
PER × ES		−0.11	0.33
FRU × ES		−0.85^**^	0.33
WS × OPE	*F*_(5, 7572.9)_ = 2.38^*^		
TEM × OPE		0.45	0.36
PHY × OPE		0.17	0.36
EFF × OPE		0.82^*^	0.36
PER × OPE		0.71^*^	0.36
FRU × OPE		0.76^*^	0.36

*B, parameter estimate; SE, standard error of the parameter estimate; RED, Redman; OSw/o, Operational Service Without Intervention; OSw, Operational Service With Intervention; TEM, Temporal Demand; PHY, Physical Demand; EFF, Effort; PER, dissatisfaction with performance; FRU, Frustration; df, degrees of freedom; EXT, Extraversion; AGR, Agreeableness; CON, Conscientiousness; ES, Emotional Stability; OPE, Openness*.

* p < 0.05;

** *p < 0.01*;

*** *p < 0.001*.

Finally, we did not find support for the hypothesis that the level of a personality trait could moderate the interaction effect of task and WS since the fit of Model 8 was not statistically higher than the fit of Model 7 [*X*^2^_(75)_ = 68.53, *p* = 0.684].

## Discussion

Seemingly for the first time, this study investigated whether personality traits moderated perceived workload, once controlling for background variables, task type and workload source, in real-life tasks of flying column police officers. Our results suggest that personality traits moderate the effect of the workload source. At a more general level, the results of this study are also consistent with the claim that workload is the result of the characteristics of the individual that interact with task characteristics (Szalma, 2008, 2009; Szalma and Teo, 2012).

Background variables did not show any significant effect (Model 1). The tasks performed by the officers of this study clearly differed in perceived workload, as suggested by the significant main effect of task (Model 2). Not surprisingly, the two most demanding tasks—regardless of the workload source—were the Redman, which is an intense training drill, and the Operational Service With Intervention, which is an emergency task that might lead to physical confrontation (see also Figure 2A). The main effect of workload source was also significant (Model 3), indicating that some workload sources, namely, Temporal Demands and Effort, are rated as more impacting than others, regardless of the task (Figure 2B). However, the significant interaction effect of task by WS (Model 4) revealed that some workload sources were more relevant for some tasks (e.g., Physical demands and Effort for the Redman task, Figure 2C).

However, the aim of this paper was to investigate the role played by personality traits in directly predicting workload levels and/or moderating the effects of the other predictors. A model that specified the main (direct) effects of personality traits (Model 5) showed that higher levels of Extraversion were associated with higher levels of perceived workload, whereas higher levels of Conscientiousness and Emotional Stability were associated with lower levels of perceived workload. These results would have been consistent with expectations only if we assumed that the tasks could not have been very stimulating for the officers, thus making them perceive the tasks as aversive (Szalma and Teo, 2012). When in Model 6 we specified the interaction of each personality trait with task type, no effect was statistically significant and only the effect of Emotional Stability was still significant among the direct effects. This result suggested that, when workload source was ruled out, personality did not moderate the effect of task type on perceived workload. The interaction of personality traits with workload sources was instead significant (Model 7) whereas the three-way interaction was not (Model 8). This result indicates that, regardless of task type, and although tasks can differ in workload scores, perceived workload seems to be a function of the interaction between a specific personality trait with a specific WS. Hence, the use of a total workload score, as it is often the case with the NASA-TLX, might conceal crucial information, at least in the population investigated in this study.

Consistent with previous studies (e.g., Rose et al., 2002), higher Neuroticism (i.e., low Emotional Stability) was associated with higher perceived frustration. This trait has been reported to be associated with negative affect (e.g., Widiger, 2009), and with higher discrepancy between performance efficiency (i.e., the amount of processing resources invested in a task) and performance effectiveness (i.e., the quality of the performance; Eysenck and Eysenck, 1985). Therefore, officers with low Emotional Stability are likely to need a higher resource and effort expenditure to reach the same level of performance of their colleagues. This might be one of the reasons for their frustration. These findings are also consistent with research suggesting that individuals high in Neuroticism adapt poorly to changing environmental conditions, such as shift work changes and sudden changes in workload levels (Akerstedt and Theorell, 1976; Cox-Fuenzalida et al., 2004). Police officers often have to perform tasks with a high degree of unpredictability, always being under public scrutiny. In response to stressful events, individuals scoring higher in Neuroticism cope less adaptively (e.g., are less task-focused and more emotion-focused) than those scoring lower in Neuroticism (e.g., Matthews et al., 2002).

Interestingly, the other personality traits also showed significant effects. Higher Agreeableness was associated with lower perceived frustration and higher perceived temporal demands. The former result is consistent with the finding that in situations of frustration individuals with higher Agreeableness strategically suppress the dominant negative responses to engage in subdominant positive interpersonal behavior (Jensen-Campbell et al., 2002). The latter result would imply that the more officers are good-natured, cooperative, forgiving, and tolerant, the more they feel the time pressure. This result seems to be consistent with previous studies that showed that higher levels of Agreeableness were associated with a decline in performance under social and time pressure, possibly because performance pressure may elicits anxiety in highly agreeable individuals (Byrne et al., 2015).

Higher Conscientiousness was associated with lower perceived temporal demands: more conscientious individuals are more likely to be more efficient and organized, and this may enable them successfully complete their tasks in less time, whence less perceived time pressure (Wayne et al., 2004).

Higher Openness was associated with higher effort, dissatisfaction with the performance, frustration and lower perception of mental demands. This result is consistent with previous studies (van Emmerik, 2008), as high Openness can be detrimental especially in work groups. Given that the task performed by the officers of this study constantly involved working in groups, those high in Openness might have experienced a reduced goal clarity (possibly because individuals high in Openness keep generating new ideas, Kickul, 2000) or a lower relationship harmony in the group (perhaps because individuals who are high in Openness tend to be individualistic, Lun and Bond, 2006). This can explain the higher workload. On the other hand, Openness seemed also to have a positive effect, as it reduced the perceived mental demands. This result is consistent with previous studies that demonstrate that high-Open individuals show greater stress resilience than low-Open individuals (Williams et al., 2009). Specifically, the lower perception of mental demands appears to be consistent with studies that found that Openness involves a propensity to be actively and flexibly engaged with novelty (particularly on an abstract, cognitive level), including finding novelty in the familiar (DeYoung et al., 2005).

Somehow unexpectedly, Extraversion did not show any significant effect. As argued by Grant and Langan-Fox (2007), in previous studies Extraversion has been linked with a positive appraisal tendency (e.g., Hemenover, 2001), and thus it can be expected to buffer the effect of perceived stress on strain regardless of stressor type. However, the positive appraisal tendency previously linked with this trait might not generalize to all stressor variables, particularly those relevant to a specific occupational context such as the one investigated in this study. Perhaps one explanation is that FCPOs should not exhibit positive affect while on duty, which causes emotional workload if their level of Extraversion is high. However, further research is needed to shed light on this issue.

In summary, the results of this study suggest that personality influence police officers' perception of workload when on duty, but only as far as different WSs are taken into account. Specifically, we provided evidence that personality may moderate the experienced workload, and a key role seems to be played especially by Agreeableness and Emotional Stability, as lower levels of these traits were associated with higher levels of frustration.

### Implications

The results of this study have several implications. When confronted with a hostile situation (e.g., a provocation), disagreeable individuals are more likely to notice, attend to, and process antisocial or hostile cues. This increases the likelihood of hostile attributions and interactions, and thereby reinforces a range of aggressive schema and scripts (Barlett and Anderson, 2012). Neuroticism (i.e., low Emotional Stability) can contribute to the etiology of a number of negative life outcomes, and, specifically, to vengefulness (McCullough et al., 2001), anger, and hostility (Sharpe and Desai, 2001). Frustration is associated with aggression and violence, possibly due to the release of catecholamine hormones such as adrenaline and noradrenaline: These hormones provide the body a burst of energy and facilitate immediate physical reactions associated with a preparation for violent muscular action (Ekkers, 1975).

Given that unnecessary violence is one of the main concerns of police officers when they are on duty, targeting the aforementioned personality characteristics in intervention programs during training for high-risk event patrolling might result not only in lower stress, and hence higher wellbeing, for officers, but, indirectly, also in less risk of police brutality for the people they have to interact with. Unfortunately, dispositional characteristics such as Agreeableness and Neuroticism have proven to be difficult to change, given their stability (e.g., Roberts and DelVecchio, 2000). Therefore, interventions aimed directly at them might not be practical or effective. However, therapeutic interventions such as cognitive behavior therapy, which specifically target dysfunctional cognitions, have been successful in treating a variety of disorders linked to anxiety, a primary component of Neuroticism (Beck, 1991), and it has been shown that counseling and psychotherapy can decrease symptoms of stress related to stressors (Bower et al., 2003).

The issue can be also tackled by training officers to become more resilient, i.e., to notice weak signals and provide strong responses to them, before they evolve toward more serious outcomes (Paton, 2006). This means enabling officers to recognize early symptoms of psychological distress, such as lack of focus and irritability, and helping them to understand that there is nothing wrong in their feelings and in seeking for professional help (Berking et al., 2010). Unfortunately, this implies a huge change in police culture, since the recognition of emotional problems is rarely encouraged in the law enforcement sector, as it is considered as a sign of weakness (Winwood et al., 2012). Personal resilience cannot be promoted without a systemic change in the organization. It is therefore necessary to promote a well-being culture, where the importance of reporting weak signals about excessive workload, stress, and burnout is widely acknowledged and is supported by reporting systems and health-promotion programs (McCraty and Atkinson, 2012).

### Limitations

Some limitations of this study need to be pointed out. First, as in any observational study in which some key variables cannot be controlled, unknown confounders might have biased the results, despite our attempt to reduce their influence through a principled statistical method, and undermined the causal interpretation of the models. However, officers were not allowed to choose the shift they had to perform, as this was determined by higher-ranked Public Security Officers. Hence, task could be considered as an exogenous variable. Moreover, personality traits are relatively stable, enduring traits with biological and heritable basis (Costa and McCrae, 1992), rendering them antecedent to workload in the work environment. Second, there was no measure of job performance, which would have provided deeper insight in the understanding of the relationships between personality, task type, and workload source. However, a quantitative and valid measure of performance might be difficult to obtain in cases like the one at hand, since performance is mainly evaluated in terms of abstaining from unnecessary violence, which has been the case for all the officers enrolled in this study. Third, the use of self-report measures of personality and workload might have been susceptible to contamination from respondent bias and/or self-presentation style (Spector and O'Connell, 1994). Actually, the availability of physiological measures of workload could have provided further insight, since subjective workload scores rated after a task may be affected by the task results, while the physiological responses recorded during the task are not (Miyake, 2001). Unfortunately, the devices available in this project could not provide reliable and valid data while being non-intrusive. Other variables, such as vigilance (Rose et al., 2002), cognitive appraisal (Cox-Fuenzalida et al., 2004), coping (Cox-Fuenzalida et al., 2004), dysfunctional cognitions (Conard and Matthews, 2008) could also have been employed as moderators, but it must be taken into account that measures of states (as opposed to traits) should have been administered after each shift together with the NASA-TLX. Moreover, the complete dataset used in this study includes measures of occupational stress, burnout, depression, anxiety and mood, which could have been entered in the LMMs as further control variables. Unfortunately, these measures were administered together with the personality questionnaire 6 months before the event, and given that they also measure states, there would have been little point in including them in the analyses. Fourth, no women officers took part to this study. Gender could have played a further moderating role since it has been found that women officers in operational service roles are more vulnerable to both organizational and operational stressors than men (Acquadro Maran et al., 2015). Fifth, we expect that the results of this study can be extended to all those police or military officer populations that have to deal with civilians in extreme contexts and whose tasks are to separate aggressively disposed civilian people within different factions without contributing to an increase in violence, to keep watch on demonstrators without letting the protests erupt in a revolt or, as it is has been recently the case in Europe, to deal with migrants at countries' borders without resorting to unnecessary violence. Hence, caution should be used in generalizing these results to non-military first responders (e.g., medical staff). Moreover, the results might have limited generalizability also due to cultural and temporal issues. The importance of personality traits varies with cultures (Williams et al., 2010). Moreover, different countries might also have different screening procedures for selecting police officers and a different public's attitude toward their conduct, which, in turn, depends on the zeitgeist and the dominant political orientation at a given time. However, police officers may experience psychological distress regardless of the outcome of the complex interaction of these factors, hence monitoring their psychological functioning and training them to identify and adequately manage emotional disturbances appears to be a successful strategy in the long run. However, more empirical evidence is needed to support this claim.

## Conclusions

The present study showed, for the first time, that FCPOs' workload response in a real-life task is the result of the interaction of their personality traits with workload sources. Since workload is one of the main causes of work-related stress, the results seem to have important implications for the prevention of this condition. One possible implication is the potential for tailoring task assignment to personality type and temperament, in order to reduce mental workload and consequently, distress. This appears to be crucial for those workers, such as police officers, whose (mis)behavior can have serious consequences on other individuals. To this aim, the NASA-TLX is a simple and efficient instrument for measuring the perceived workload and its application can be useful in monitoring the workers assigned to critical tasks.

### Conflict of interest statement

The authors declare that the research was conducted in the absence of any commercial or financial relationships that could be construed as a potential conflict of interest.

## Footnotes

^1^Decision latitude is “the ability to make work-related decisions. When employees can make decisions related to the way they work, they are able to devise coping strategies than can mitigate the effects of stress” (Halpern, 2005, p. 159).

^2^A relatively small, independent, police unit capable of rapid mobility.

^3^The cohort also includes two women officers, but they were not on duty during the event in which NASA-TLX scores were collected.
