# Supplementary material for: Personality Traits Moderate the Effect of Workload Sources on Perceived Workload in Flying Column Police Officers
Source: Front Psychol. 2015 Nov 27;6:1835. doi: 10.3389/fpsyg.2015.01835 (PMC4661321; doi:10.3389/fpsyg.2015.01835)
Supplement: Supplementary file 1 [file DataSheet1.DOC]

Supplementary Material

# Personality traits moderate the effect of real-life tasks on perceived workload in flying column police officers

Carlo Chiorri*, Sergio Garbarino, Fabrizio Bracco, Nicola Magnavita

*** Correspondence:** Carlo Chiorri: carlo.chiorri@unige.it

# Models that account for the role played by individual differences in stress response

The differential exposure model assumes that individual differences influence exposure to stressors by selecting individuals to situations where they are exposed to certain stressors, e.g. work characteristics (Bolger and Zuckerman, 1995). The differential reactivity model proposes that individual differences affect stress reactivity: hence, the effect of a change in stressors on workers' well-being depends on their personality (Bolger and Zuckerman, 1995). The differential exposure-reactivity model combines the two previous models and thus hypothesizes that individual differences impact both exposure and response to stressors (Bolger and Zuckerman, 1995). A model that assumes that stressors influence personality, either directly or through well-being, has also been proposed ('outcome model') (Kivimäki, 1996). According to Hancock and Warm's dynamic adaptability theory of performance under stress (Hancock and Warm, 1989), the spatial organization of a task (information structure) and the temporal properties of a task (information rate) are the most proximal sources of workload and stress input. Individuals can maintain a stable adaptive state across a wide range of task demands, but, when these reach extremes, workload and stress increase and performance impairments can occur. Szalma (2008) suggested that models of stress and performance should include both person and task characteristics to fully account for workload and stress effects, since stress results from an interaction between the individual and the task (Matthews, 2001).

# Review of studies that investigated personality traits in flying column police officers

The Italian Ministry of Interior developed a program to monitor stress conditions of the officers of a flying column, the VI Reparto Mobile in Genoa. Officers underwent a detailed psychological assessment that included measures of personality, depression, state anxiety, mood stability, burn-out and work-related stress, along with recordings of clinical outcomes (e.g., quantification of short-term sickness absences). This led to the development of a large database containing all the data collected during the project, which can be accessed under authorization of the Ministry of Interior

A few studies have already been published using data from this database (e.g., Garbarino et al., 2011a, 2011b, 2013, Magnavita and Garbarino, 2013), but only some of them included the available measures of the Five-Factor Model (FFM) personality traits. Garbarino et al. (2012a) applied cluster analysis to personality scores and found that officers could be categorized into two robust profile groups, that were labelled as Resilient (32.2%) and Undercontrolled (67.8%). The officers in the former group showed higher levels of Extraversion (i.e., being sociable, gregarious, assertive, talkative, and dynamic), Agreeableness (i.e., being courteous, flexible, trusting, good-natured, cooperative, forgiving, and tolerant), Conscientiousness (i.e., being dependable, hard-working, achievement-oriented, and persevering) and Openness (i.e., being imaginative, cultured, curious, original, broad-minded, intelligent, and artistically sensitive) and lower levels of Neuroticism (i.e., being anxious, depressed, emotionally unstable, worried, and insecure) than the officers in the latter. Moreover, they reported lower levels of depression, anxiety, professional exhaustion, loss of empathy, and endorsed higher scores in a measure of perceived organizational resilience. Though the clusters did not differ in mood stability, the Resilient officers reported significantly lower levels of negative mood and higher levels of vigor-activity. More recently, Garbarino et al. (2014) found that higher levels of Neuroticism were associated with higher levels of job strain (i.e., perceived imbalance between job demands and job decision latitude) and of effort/reward imbalance (i.e., perceived imbalance between the efforts spent on the job and the rewards received). Besides, higher levels of Agreeableness were associated with lower levels effort/reward imbalance. In another study carried out before and during the 2009 35th G8 summit meeting held in L’Aquila Garbarino et al. (2012b) showed that during the event Resilient officers reported lower stress levels and had fewer short-term sickness absences than during regular duty.

# References for these Supplementary Materials

Bolger, N., and Zuckerman, A. (1995). A framework for studying personality in the stress process. *J. Pers. Soc. Psychol.* 69, 890–902. doi:10.1037/0022-3514.69.5.890.

Garbarino, S., Chiorri, C., Magnavita, N., Piattino, S., and Cuomo, G. (2012a). Personality profiles of special force police officers. *J. Police Crim. Psychol.* 27, 99–110. doi:10.1007/s11896-011-9099-6.

Garbarino, S., Cuomo, G., Chiorri, C., and Magnavita, N. (2013). Association of work-related stress with mental health problems in a special police force unit. *BMJ Open* 3, 1–12. doi:10.1136/bmjopen-2013-002791.

Garbarino, S., Magnavita, N., and Chiorri, C. (2014). Personality traits of the Five-Factor Model are associated with work-related stress in special force police officers. *Int. Arch. Occup. Environ. Health* 87, 295–306. doi:10.1007/s00420-013-0861-1.

Garbarino, S., Magnavita, N., Chiorri, C., Brisinda, D., Cuomo, G., Venuti, A., and Fenici, R. (2012b). Evaluation of operational stress in riot and crowd control police units: A global challenge for prevention and management of police task-related stress. *J. Police Crim. Psychol.* 27, 111–122. doi:10.1007/s11896-012-9104-8.

Garbarino, S., Magnavita, N., Ciprani, F., and Cuomo, G. (2011b). The coping and support activities in the phenomenon of mass immigration in Italy: evaluation of work-related stress in police forces. *G. Ital. Med. Lav. Ergon.* 33(3 Suppl), 170–174.

Garbarino, S., Magnavita, N., Elovainio, M., Heponiemi, T., Ciprani, F., Cuomo, G., and Bergamaschi, A. (2011a). Police job strain during routine activities and a major event. *Occup. Med. (Chic. Ill).* 61, 395–399. doi:10.1093/occmed/kqr058.

Hancock, P. A., and Warm, J. S. (1989). A dynamic model of stress and sustained attention. *Hum. Factors* 31, 519–537. doi:10.1177/001872088903100503.

Kivimäki, M. (1996). *Stress and personality factors: specifications of the role of test anxiety, private self-consciousness, type A behavior pattern, and self-esteem in the relationship between stressors and stress reactions*. Helsinki: Finnish Institute of Occupational Health.

Magnavita, N., and Garbarino, S. (2013). Is absence related to work stress? A repeated cross-sectional study on a special police force. *Am. J. Ind. Med.* 56, 765–775. doi:10.1002/ajim.22155.

Matthews, G. (2001). “Levels of transaction: A cognitive science framework for operator stress.,” in *Stress, workload, and fatigue.* Human factors in transportation., eds. P. A. Hancock and P. A. Desmond (Lawrence Erlbaum Associates Publishers), 5–33. Available at: http://search.ebscohost.com/login.aspx?direct=true&db=psyh&AN=2000-14014-001&site=ehost-live.

Szalma, J. L. (2008). “Individual differences in stress reaction,” in *Performance under stress*, eds. P. A. Hancock and J. L. Szalma (Aldershot, UK: Ashgate), 323–357.

# Supplementary Tables: Background characteristics of the police officers enrolled in the study

| Variable | Statistic |
| --- | --- |
| Age (years, M±SD, range) | 35.03±7.60, 23-56 |
| Marital Status |  |
| Single | 59.5% |
| Married | 34.6% |
| Divorced | 5.9% |
| Having children | 33.5% |
| Resident in the region where the unit was based | 27.0% |
| Educational level |  |
| Low (less than high school) | 23.8% |
| Medium (high school, 13 years of education) | 72.9% |
| High (more than high school) | 3.3% |
| Role |  |
| Agents ('*agente*' or '*assistente*') | 86.3% |
| Heads ('*sovrintendente*' or '*ispettore*') | 7.8% |
| Technician | 5.9% |
| Years in service (M±SD, range) | 13.85±7.94, 3-37 |
| Being quartered in barracks | 59.1% |

# Supplementary Tables: Results of hierarchical linear mixed-effects models estimating the effects of task, workload source, personality traits and their interaction on perceived workload of special force police officers

|  | Model 1 | | |  | Model 2 | | |
| --- | --- | --- | --- | --- | --- | --- | --- |
|  | Effect | B | SE |  | Effect | B | SE |
| Age |  | 0.05 | 0.03 |  |  | 0.04 | 0.03 |
| Marital Status | F(2,276.8)=1.06 |  |  |  | F(2,273.0)=1.39 |  |  |
| Married |  | -0.14 | 0.36 |  |  | -0.09 | 0.36 |
| Divorced |  | 0.58 | 0.52 |  |  | 0.71 | 0.52 |
| Having children |  | -0.09 | 0.37 |  |  | -0.05 | 0.37 |
| Residence in the region |  | -0.23 | 0.27 |  |  | -0.18 | 0.26 |
| Education | F(2,258.3)=0.17 |  |  |  | F(2,255.8)=0.13 |  |  |
| Medium |  | -0.19 | 0.34 |  |  | -0.15 | 0.34 |
| High |  | -0.09 | 0.66 |  |  | 0.01 | 0.66 |
| Role | F(2,291.2)=1.11 |  |  |  | F(2,286.6)=1.10 |  |  |
| Heads |  | -0.50 | 0.48 |  |  | -0.46 | 0.47 |
| Technician |  | 0.35 | 0.59 |  |  | 0.38 | 0.58 |
| Years in service |  | -0.01 | 0.04 |  |  | -0.01 | 0.04 |
| Being quartered in barracks |  | 0.03 | 0.30 |  |  | 0.06 | 0.30 |
| Task |  |  |  |  | F(3,7801.6)=256.41*** |  |  |
| RED |  |  |  |  |  | 3.59*** | 0.23 |
| OSw/o |  |  |  |  |  | -3.03*** | 0.22 |
| OSw |  |  |  |  |  | 2.13*** | 0.24 |
|  |  |  |  |  |  |  |  |
| df | 14 |  |  |  | 17 |  |  |
| Δdf | 11 |  |  |  | 3 |  |  |
| AIC | 54507.23 |  |  |  | 53780.08 |  |  |
| BIC | 54604.77 |  |  |  | 53898.53 |  |  |
| LL | -27239.61 |  |  |  | -26873.04 |  |  |
| Deviance | 54479.23 |  |  |  | 53746.08 |  |  |
| ΔDeviance | 15.93 |  |  |  | 733.14*** |  |  |

(continues)

Note: B = parameter estimate; SE = standard error of the parameter estimate; RED = Redman; OSw/o: Operational Service Without Intervention; OSw = Operational Service With Intervention; df = degrees of freedom; Δdf = difference in degrees of freedom with respect to the previous model; ACI = Akaike Information Criterion; BIC = Schwarz's Bayesian Information Criterion; LL=Log-likelihood; ΔDeviance = difference in degrees of freedom with respect to the previous model.

* = *p* < .05; ** = *p* < .01; *** = *p* < .001.

|  | Model 3 | | |  | Model 4 | | |
| --- | --- | --- | --- | --- | --- | --- | --- |
|  | Effect | B | SE |  | Effect | B | SE |
| Age |  | 0.04 | 0.03 |  |  | 0.04 | 0.03 |
| Marital Status | F(2,271.8)=1.39 |  |  |  | F(2,270.4)=1.39 |  |  |
| Married |  | -0.09 | 0.36 |  |  | -0.10 | 0.36 |
| Divorced |  | 0.71 | 0.52 |  |  | 0.70 | 0.52 |
| Having children |  | -0.05 | 0.37 |  |  | -0.05 | 0.37 |
| Residence in the region |  | -0.18 | 0.26 |  |  | -0.18 | 0.26 |
| Education | F(2,255.8)=0.12 |  |  |  | F(2,256)=0.11 |  |  |
| Medium |  | -0.14 | 0.34 |  |  | -0.14 | 0.34 |
| High |  | 0.01 | 0.66 |  |  | 0.01 | 0.66 |
| Role | F(2,284.9)=1.12 |  |  |  | F(2,282.5)=1.14 |  |  |
| Heads |  | -0.47 | 0.47 |  |  | -0.48 | 0.47 |
| Technician |  | 0.37 | 0.58 |  |  | 0.36 | 0.58 |
| Years in service |  | -0.01 | 0.04 |  |  | -0.01 | 0.04 |
| Being quartered in barracks |  | 0.06 | 0.30 |  |  | 0.06 | 0.30 |
| Task | F(3,7795.2)=276.24*** |  |  |  | F(3,7783.9)=309.47*** |  |  |
| RED |  | 3.59*** | 0.22 |  |  | 3.95*** | 0.51 |
| OSw/o |  | -3.03*** | 0.22 |  |  | -1.50** | 0.50 |
| OSw |  | 2.13*** | 0.23 |  |  | 1.08* | 0.52 |
| Workload Source | F(5,7575.9)=118.14*** |  |  |  | F(5,7574.3)=157.84*** |  |  |
| TEM |  | 5.76*** | 0.28 |  |  | 4.83*** | 0.43 |
| PHY |  | 1.54*** | 0.28 |  |  | 2.09*** | 0.43 |
| EFF |  | 2.82*** | 0.28 |  |  | 1.59*** | 0.43 |
| PER |  | 0.99*** | 0.28 |  |  | 1.71*** | 0.43 |
| FRU |  | 0.29 | 0.28 |  |  | 2.49*** | 0.43 |
| Task x Workload Source |  |  |  |  | F(15,7574.3)=61.17*** |  |  |
| RED x TEM |  |  |  |  |  | 6.08*** | 0.73 |
| OSw/o x TEM |  |  |  |  |  | -4.75*** | 0.70 |
| OSw x TEM |  |  |  |  |  | 3.95*** | 0.73 |
| RED x PHY |  |  |  |  |  | -3.33*** | 0.73 |
| OSw/o x PHY |  |  |  |  |  | -1.18 | 0.70 |
| OSw x PHY |  |  |  |  |  | 2.04** | 0.73 |
| RED x EFF |  |  |  |  |  | 5.47*** | 0.73 |
| OSw/o x EFF |  |  |  |  |  | -2.22** | 0.70 |
| OSw x EFF |  |  |  |  |  | 3.20*** | 0.73 |
| RED x PER |  |  |  |  |  | -2.22** | 0.73 |
| OSw/o x PER |  |  |  |  |  | 0.28 | 0.70 |
| OSw x PER |  |  |  |  |  | -1.68* | 0.73 |
| RED x FRU |  |  |  |  |  | -8.16*** | 0.73 |
| OSw/o x FRU |  |  |  |  |  | -1.33 | 0.70 |
| OSw x FRU |  |  |  |  |  | -1.20 | 0.73 |
|  |  |  |  |  |  |  |  |
| df | 22 |  |  |  | 37 |  |  |
| Δdf | 5 |  |  |  | 15 |  |  |
| AIC | 53221.27 |  |  |  | 52385.14 |  |  |
| BIC | 53374.55 |  |  |  | 52642.93 |  |  |
| LL | -26588.64 |  |  |  | -26155.57 |  |  |
| Deviance | 53177.27 |  |  |  | 52311.14 |  |  |
| ΔDeviance | 568.81*** |  |  |  | 866.13*** |  |  |

Note: B = parameter estimate; SE = standard error of the parameter estimate; RED = Redman; OSw/o: Operational Service Without Intervention; OSw = Operational Service With Intervention; TEM = Temporal Demand; PHY = Physical Demand; EFF = Effort; PER = dissatisfaction with performance; FRU = Frustration; df = degrees of freedom; Δdf = difference in degrees of freedom with respect to the previous model; ACI = Akaike Information Criterion; BIC = Schwarz's Bayesian Information Criterion; LL=Log-likelihood; ΔDeviance = difference in degrees of freedom with respect to the previous model.; * = *p* < .05; ** = *p* < .01; *** = *p* < .001.

|  | Model 5 | | |  | Model 6 | | |
| --- | --- | --- | --- | --- | --- | --- | --- |
|  | Effect | B | SE |  | Effect | B | SE |
| Age |  | 0.04 | 0.03 |  |  | 0.04 | 0.03 |
| Marital Status | F(2,270.2)=0.98 |  |  |  | F(2,270)=0.95 |  |  |
| Married |  | 0.00 | 0.35 |  |  | 0.00 | 0.35 |
| Divorced |  | 0.64 | 0.51 |  |  | 0.63 | 0.51 |
| Having children |  | 0.03 | 0.36 |  |  | 0.04 | 0.36 |
| Residence in the region |  | -0.14 | 0.26 |  |  | -0.14 | 0.26 |
| Education | F(2,254.6)=0.12 |  |  |  | F(2,254.5)=0.12 |  |  |
| Medium |  | -0.09 | 0.34 |  |  | -0.09 | 0.34 |
| High |  | 0.13 | 0.65 |  |  | 0.13 | 0.65 |
| Role | F(2,282.1)=1.73 |  |  |  | F(2,282)=1.77 |  |  |
| Heads |  | -0.79. | 0.47 |  |  | -0.79. | 0.47 |
| Technician |  | 0.01 | 0.57 |  |  | 0.03 | 0.57 |
| Years in service |  | -0.01 | 0.03 |  |  | -0.01 | 0.03 |
| Being quartered in barracks |  | 0.11 | 0.29 |  |  | 0.12 | 0.29 |
| Task | F(3,7788.1)=309.12*** |  |  |  | F(3,7788.4)=303.06*** |  |  |
| RED |  | 3.94*** | 0.51 |  |  | 3.95*** | 0.51 |
| OSw/o |  | -1.51** | 0.50 |  |  | -1.47** | 0.50 |
| OSw |  | 1.07* | 0.52 |  |  | 1.11* | 0.52 |
| Workload Source | F(5,7573.7)=157.83*** |  |  |  | F(5,7573.1)=158.08*** |  |  |
| TEM |  | 4.83*** | 0.43 |  |  | 4.83*** | 0.43 |
| PHY |  | 2.09*** | 0.43 |  |  | 2.09*** | 0.43 |
| EFF |  | 1.59*** | 0.43 |  |  | 1.59*** | 0.43 |
| PER |  | 1.71*** | 0.43 |  |  | 1.71*** | 0.43 |
| FRU |  | 2.49*** | 0.43 |  |  | 2.49*** | 0.43 |
| Task x Workload Source | F(15,7573.7)=61.17*** |  |  |  | F(15,7573.1)=61.26*** |  |  |
| RED x TEM |  | 6.08*** | 0.73 |  |  | 6.08*** | 0.73 |
| OSw/o x TEM |  | -4.75*** | 0.70 |  |  | -4.75*** | 0.70 |
| OSw x TEM |  | 3.95*** | 0.73 |  |  | 3.95*** | 0.73 |
| RED x PHY |  | -3.33*** | 0.73 |  |  | -3.33*** | 0.73 |
| OSw/o x PHY |  | -1.18. | 0.70 |  |  | -1.18. | 0.70 |
| OSw x PHY |  | 2.04** | 0.73 |  |  | 2.04** | 0.73 |
| RED x EFF |  | 5.47*** | 0.73 |  |  | 5.47*** | 0.73 |
| OSw/o x EFF |  | -2.22** | 0.70 |  |  | -2.22** | 0.70 |
| OSw x EFF |  | 3.20*** | 0.73 |  |  | 3.20*** | 0.73 |
| RED x PER |  | -2.22** | 0.73 |  |  | -2.22** | 0.73 |
| OSw/o x PER |  | 0.28 | 0.70 |  |  | 0.28 | 0.70 |
| OSw x PER |  | -1.68* | 0.73 |  |  | -1.68* | 0.73 |
| RED x FRU |  | -8.16*** | 0.73 |  |  | -8.16*** | 0.73 |
| OSw/o x FRU |  | -1.33. | 0.70 |  |  | -1.33. | 0.70 |
| OSw x FRU |  | -1.20 | 0.73 |  |  | -1.20 | 0.73 |
| Personality traits |  |  |  |  |  |  |  |
| EXT |  | 0.25* | 0.13 |  |  | 0.21 | 0.18 |
| AGR |  | 0.22 | 0.14 |  |  | 0.21 | 0.20 |
| CON |  | -0.28* | 0.12 |  |  | -0.06 | 0.17 |
| ES |  | -0.33* | 0.13 |  |  | -0.59*** | 0.18 |
| OPE |  | -0.11 | 0.15 |  |  | 0.02 | 0.20 |
| Task x EXT |  |  |  |  | F(3,7768.2)=0.48 |  |  |
| RED x EXT |  |  |  |  |  | -0.12 | 0.26 |
| OSw/o x EXT |  |  |  |  |  | 0.21 | 0.26 |
| OSw x EXT |  |  |  |  |  | 0.12 | 0.26 |
| Task x AGR |  |  |  |  | F(3,7756.4)=0.06 |  |  |
| RED x AGR |  |  |  |  |  | -0.04 | 0.29 |
| OSw/o x AGR |  |  |  |  |  | 0.08 | 0.28 |
| OSw x AGR |  |  |  |  |  | -0.05 | 0.30 |
| Task x CON |  |  |  |  | F(3,7778.2)=1.43 |  |  |
| RED x CON |  |  |  |  |  | -0.22 | 0.26 |
| OSw/o x CON |  |  |  |  |  | -0.50 | 0.25 |
| OSw x CON |  |  |  |  |  | -0.29 | 0.25 |
| Task x ES |  |  |  |  | F(3,7798.1)=1.54 |  |  |
| RED x ES |  |  |  |  |  | 0.37 | 0.26 |
| OSw/o x ES |  |  |  |  |  | 0.37 | 0.25 |
| OSw x ES |  |  |  |  |  | 0.51. | 0.27 |
| Task x OPE |  |  |  |  | F(3,7766.9)=0.66 |  |  |
| RED x OPE |  |  |  |  |  | -0.05 | 0.29 |
| OSw/o x OPE |  |  |  |  |  | -0.30 | 0.28 |
| OSw x OPE |  |  |  |  |  | -0.32 | 0.29 |
|  |  |  |  |  |  |  |  |
| df | 42 |  |  |  | 57 |  |  |
| Δdf | 5 |  |  |  | 15 |  |  |
| AIC | 52378.67 |  |  |  | 52397.19 |  |  |
| BIC | 52671.30 |  |  |  | 52794.33 |  |  |
| LL | -26147.34 |  |  |  | -26141.6 |  |  |
| Deviance | 52294.67 |  |  |  | 52283.19 |  |  |
| ΔDeviance | 16.47** |  |  |  | 11.48 |  |  |

(continues)

Note: B = parameter estimate; SE = standard error of the parameter estimate; RED = Redman; OSw/o: Operational Service Without Intervention; OSw = Operational Service With Intervention; TEM = Temporal Demand; PHY = Physical Demand; EFF = Effort; PER = dissatisfaction with performance; FRU = Frustration; df = degrees of freedom; EXT = Extraversion; AGR = Agreeableness; CON = Conscientiousness; ES = Emotional Stability; OPE = Openness; Δdf = difference in degrees of freedom with respect to the previous model; ACI = Akaike Information Criterion; BIC = Schwarz's Bayesian Information Criterion; LL=Log-likelihood; ΔDeviance = difference in degrees of freedom with respect to the previous model.

* = *p* < .05; ** = *p* < .01; *** = *p* < .001.

|  | Model 7 |  |  |  | Model 8 |  |  |
| --- | --- | --- | --- | --- | --- | --- | --- |
|  | Effect | B | SE |  | Effect | B | SE |
| Age |  | 0.04 | 0.03 |  |  | 0.04 | 0.03 |
| Marital Status | F(2,269.8)=0.95 |  |  |  | F(2,269.7)=0.95 |  |  |
| Married |  | 0.00 | 0.35 |  |  | 0.00 | 0.35 |
| Divorced |  | 0.63 | 0.51 |  |  | 0.63 | 0.51 |
| Having children |  | 0.04 | 0.36 |  |  | 0.04 | 0.36 |
| Residence in the region |  | -0.14 | 0.26 |  |  | -0.14 | 0.26 |
| Education | F(2,254.5)=0.12 |  |  |  | F(2,254.6)=0.12 |  |  |
| Medium |  | -0.09 | 0.34 |  |  | -0.09 | 0.34 |
| High |  | 0.13 | 0.65 |  |  | 0.13 | 0.65 |
| Role | F(2,281.8)=1.78 |  |  |  | F(2,281.6)=1.78 |  |  |
| Heads |  | -0.79. | 0.47 |  |  | -0.79. | 0.47 |
| Technician |  | 0.03 | 0.57 |  |  | 0.03 | 0.57 |
| Years in service |  | -0.01 | 0.03 |  |  | -0.01 | 0.03 |
| Being quartered in barracks |  | 0.12 | 0.29 |  |  | 0.12 | 0.29 |
| Task | F(3,7787.4)=305.97*** |  |  |  | F(3,7786.5)=308.74*** |  |  |
| RED |  | 3.98*** | 0.51 |  |  | 4.07*** | 0.51 |
| OSw/o |  | -1.46** | 0.49 |  |  | -1.48** | 0.49 |
| OSw |  | 1.13* | 0.52 |  |  | 1.18* | 0.52 |
| Workload Source | F(5,7572.9)=155.72*** |  |  |  | F(5,7572.8)=156.09*** |  |  |
| TEM |  | 4.82*** | 0.43 |  |  | 4.84*** | 0.43 |
| PHY |  | 2.08*** | 0.43 |  |  | 2.05*** | 0.43 |
| EFF |  | 1.55*** | 0.43 |  |  | 1.56*** | 0.43 |
| PER |  | 1.69*** | 0.43 |  |  | 1.71*** | 0.43 |
| FRU |  | 2.47*** | 0.43 |  |  | 2.54*** | 0.43 |
| Task x Workload Source | F(15,7572.9)=61.36*** |  |  |  | F(15,7572.8)=60.55*** |  |  |
| RED x TEM |  | 6.01*** | 0.72 |  |  | 5.85*** | 0.73 |
| OSw/o x TEM |  | -4.77*** | 0.70 |  |  | -4.74*** | 0.70 |
| OSw x TEM |  | 3.90*** | 0.73 |  |  | 3.88*** | 0.73 |
| RED x PHY |  | -3.34*** | 0.72 |  |  | -3.29*** | 0.73 |
| OSw/o x PHY |  | -1.18. | 0.70 |  |  | -1.12 | 0.70 |
| OSw x PHY |  | 2.03** | 0.73 |  |  | 2.03** | 0.73 |
| RED x EFF |  | 5.41*** | 0.72 |  |  | 5.28*** | 0.73 |
| OSw/o x EFF |  | -2.23** | 0.70 |  |  | -2.18** | 0.70 |
| OSw x EFF |  | 3.16*** | 0.73 |  |  | 3.11*** | 0.73 |
| RED x PER |  | -2.25** | 0.72 |  |  | -2.35** | 0.73 |
| OSw/o x PER |  | 0.26 | 0.70 |  |  | 0.28 | 0.70 |
| OSw x PER |  | -1.71* | 0.73 |  |  | -1.75* | 0.73 |
| RED x FRU |  | -8.17*** | 0.72 |  |  | -8.39*** | 0.73 |
| OSw/o x FRU |  | -1.36. | 0.70 |  |  | -1.37* | 0.70 |
| OSw x FRU |  | -1.19 | 0.73 |  |  | -1.38. | 0.73 |
| Personality traits |  |  |  |  |  |  |  |
| EXT |  | 0.12 | 0.28 |  |  | 0.04 | 0.39 |
| AGR |  | 0.32 | 0.31 |  |  | 0.17 | 0.44 |
| CON |  | 0.22 | 0.27 |  |  | 0.17 | 0.38 |
| ES |  | -0.31 | 0.28 |  |  | -0.05 | 0.39 |
| OPE |  | -0.46 | 0.30 |  |  | -0.25 | 0.42 |
| Task x EXT | F(3,7767.4)=0.49 |  |  |  | F(3,7766.5)=0.49 |  |  |
| RED x EXT |  | -0.12 | 0.26 |  |  | -0.42 | 0.64 |
| OSw/o x EXT |  | 0.21 | 0.26 |  |  | 0.19 | 0.62 |
| OSw x EXT |  | 0.12 | 0.26 |  |  | 0.80 | 0.63 |
| Task x AGR | F(3,7755.3)=0.07 |  |  |  | F(3,7754.3)=0.07 |  |  |
| RED x AGR |  | -0.04 | 0.29 |  |  | -0.01 | 0.71 |
| OSw/o x AGR |  | 0.08 | 0.28 |  |  | 0.68 | 0.68 |
| OSw x AGR |  | -0.05 | 0.30 |  |  | -0.09 | 0.73 |
| Task x CON | F(3,7777.2)=1.45 |  |  |  | F(3,7776.2)=1.46 |  |  |
| RED x CON |  | -0.22 | 0.25 |  |  | 0.03 | 0.62 |
| OSw/o x CON |  | -0.50 | 0.24 |  |  | -0.40 | 0.59 |
| OSw x CON |  | -0.29 | 0.25 |  |  | -0.36 | 0.61 |
| Task x ES | F(3,7797.1)=1.56 |  |  |  | F(3,7796.2)=1.58 |  |  |
| RED x ES |  | 0.37 | 0.26 |  |  | -0.18 | 0.64 |
| OSw/o x ES |  | 0.37 | 0.25 |  |  | -0.15 | 0.61 |
| OSw x ES |  | 0.51. | 0.27 |  |  | 0.38 | 0.65 |
| Task x OPE | F(3,7765.8)=0.66 |  |  |  | F(3,7764.7)=0.67 |  |  |
| RED x OPE |  | -0.05 | 0.29 |  |  | -0.88 | 0.70 |
| OSw/o x OPE |  | -0.30 | 0.28 |  |  | -0.09 | 0.68 |
| OSw x OPE |  | -0.32 | 0.29 |  |  | -0.77 | 0.69 |
| Workload Source x EXT | F(5,7572.9)=0.48 |  |  |  | F(5,7572.8)=0.25 |  |  |
| TEM x EXT |  | 0.11 | 0.33 |  |  | 0.18 | 0.53 |
| PHY x EXT |  | -0.02 | 0.33 |  |  | -0.12 | 0.53 |
| EFF x EXT |  | -0.02 | 0.33 |  |  | 0.05 | 0.53 |
| PER x EXT |  | 0.04 | 0.33 |  |  | 0.07 | 0.53 |
| FRU x EXT |  | 0.40 | 0.33 |  |  | 0.83 | 0.53 |
| Workload Source x AGR | F(5,7572.9)=5.88*** |  |  |  | F(5,7572.8)=5.55*** |  |  |
| TEM x AGR |  | 0.81* | 0.37 |  |  | 0.98 | 0.60 |
| PHY x AGR |  | 0.00 | 0.37 |  |  | 0.14 | 0.60 |
| EFF x AGR |  | 0.06 | 0.37 |  |  | 0.18 | 0.60 |
| PER x AGR |  | -0.40 | 0.37 |  |  | 0.18 | 0.60 |
| FRU x AGR |  | -1.11** | 0.37 |  |  | -1.22* | 0.60 |
| Workload Source x CON | F(5,7572.9)=2.34* |  |  |  | F(5,7572.8)=2.32* |  |  |
| TEM x CON |  | -0.74* | 0.31 |  |  | -0.66* | 0.52 |
| PHY x CON |  | -0.10 | 0.31 |  |  | -0.19 | 0.52 |
| EFF x CON |  | -0.55 | 0.31 |  |  | -0.53 | 0.52 |
| PER x CON |  | -0.45 | 0.31 |  |  | -0.68 | 0.52 |
| FRU x CON |  | 0.12 | 0.31 |  |  | 0.64 | 0.52 |
| Workload Source x ES | F(5,7572.9)=2.44* |  |  |  | F(5,7572.8)=2.41* |  |  |
| TEM x ES |  | -0.41 | 0.33 |  |  | -0.76 | 0.53 |
| PHY x ES |  | -0.04 | 0.33 |  |  | 0.06 | 0.53 |
| EFF x ES |  | -0.27 | 0.33 |  |  | -0.39 | 0.53 |
| PER x ES |  | -0.11 | 0.33 |  |  | -0.47 | 0.53 |
| FRU x ES |  | -0.85** | 0.33 |  |  | -1.67** | 0.53 |
| Workload Source x OPE | F(5,7572.9)=2.38* |  |  |  | F(5,7572.8)=2.36* |  |  |
| TEM x OPE |  | 0.45 | 0.36 |  |  | 0.25 | 0.57 |
| PHY x OPE |  | 0.17 | 0.36 |  |  | 0.44 | 0.57 |
| EFF x OPE |  | 0.82* | 0.36 |  |  | 0.67 | 0.57 |
| PER x OPE |  | 0.71* | 0.36 |  |  | 0.48 | 0.57 |
| FRU x OPE |  | 0.76* | 0.36 |  |  | -0.19 | 0.57 |
| Task x Scale x EXT |  |  |  |  | F(15,7572.8)=0.62 |  |  |
| RED x TEM x EXT |  |  |  |  |  | -0.31 | 0.90 |
| OSw/o x TEM x EXT |  |  |  |  |  | 0.27 | 0.88 |
| OSw x TEM x EXT |  |  |  |  |  | -0.32 | 0.89 |
| RED x PHY x EXT |  |  |  |  |  | 0.77 | 0.90 |
| OSw/o x PHY x EXT |  |  |  |  |  | 0.43 | 0.88 |
| OSw x PHY x EXT |  |  |  |  |  | -0.63 | 0.89 |
| RED x EFF x EXT |  |  |  |  |  | 0.34 | 0.90 |
| OSw/o x EFF x EXT |  |  |  |  |  | 0.02 | 0.88 |
| OSw x EFF x EXT |  |  |  |  |  | -0.62 | 0.89 |
| RED x PER x EXT |  |  |  |  |  | 0.75 | 0.90 |
| OSw/o x PER x EXT |  |  |  |  |  | 0.15 | 0.88 |
| OSw x PER x EXT |  |  |  |  |  | -0.93 | 0.89 |
| RED x FRU x EXT |  |  |  |  |  | 0.26 | 0.90 |
| OSw/o x FRU x EXT |  |  |  |  |  | -0.75 | 0.88 |
| OSw x FRU x EXT |  |  |  |  |  | -1.60. | 0.89 |
| Task x Scale x AGR |  |  |  |  | F(15,7572.8)=0.95 |  |  |
| RED x TEM x AGR |  |  |  |  |  | 0.34 | 1.00 |
| OSw/o x TEM x AGR |  |  |  |  |  | -1.10 | 0.96 |
| OSw x TEM x AGR |  |  |  |  |  | 0.12 | 1.03 |
| RED x PHY x AGR |  |  |  |  |  | -0.22 | 1.00 |
| OSw/o x PHY x AGR |  |  |  |  |  | -0.46 | 0.96 |
| OSw x PHY x AGR |  |  |  |  |  | 0.17 | 1.03 |
| RED x EFF x AGR |  |  |  |  |  | 0.87 | 1.00 |
| OSw/o x EFF x AGR |  |  |  |  |  | -1.30 | 0.96 |
| OSw x EFF x AGR |  |  |  |  |  | 0.08 | 1.03 |
| RED x PER x AGR |  |  |  |  |  | -0.80 | 1.00 |
| OSw/o x PER x AGR |  |  |  |  |  | -0.74 | 0.96 |
| OSw x PER x AGR |  |  |  |  |  | -1.26 | 1.03 |
| RED x FRU x AGR |  |  |  |  |  | -0.37 | 1.00 |
| OSw/o x FRU x AGR |  |  |  |  |  | -0.04 | 0.96 |
| OSw x FRU x AGR |  |  |  |  |  | 1.10 | 1.03 |
| Task x Scale x CON |  |  |  |  | F(15,7572.8)=0.52 |  |  |
| RED x TEM x CON |  |  |  |  |  | -0.24 | 0.87 |
| OSw/o x TEM x CON |  |  |  |  |  | 0.08 | 0.84 |
| OSw x TEM x CON |  |  |  |  |  | -0.24 | 0.86 |
| RED x PHY x CON |  |  |  |  |  | 0.17 | 0.87 |
| OSw/o x PHY x CON |  |  |  |  |  | 0.09 | 0.84 |
| OSw x PHY x CON |  |  |  |  |  | 0.10 | 0.86 |
| RED x EFF x CON |  |  |  |  |  | -0.40 | 0.87 |
| OSw/o x EFF x CON |  |  |  |  |  | 0.26 | 0.84 |
| OSw x EFF x CON |  |  |  |  |  | -0.04 | 0.86 |
| RED x PER x CON |  |  |  |  |  | -0.08 | 0.87 |
| OSw/o x PER x CON |  |  |  |  |  | 0.09 | 0.84 |
| OSw x PER x CON |  |  |  |  |  | 1.01 | 0.86 |
| RED x FRU x CON |  |  |  |  |  | -0.94 | 0.87 |
| OSw/o x FRU x CON |  |  |  |  |  | -1.10 | 0.84 |
| OSw x FRU x CON |  |  |  |  |  | -0.40 | 0.86 |
| Task x Scale x ES |  |  |  |  | F(15,7572.8)=0.96 |  |  |
| RED x TEM x ES |  |  |  |  |  | 0.58 | 0.90 |
| OSw/o x TEM x ES |  |  |  |  |  | 0.56 | 0.86 |
| OSw x TEM x ES |  |  |  |  |  | 0.53 | 0.92 |
| RED x PHY x ES |  |  |  |  |  | 0.27 | 0.90 |
| OSw/o x PHY x ES |  |  |  |  |  | -0.20 | 0.86 |
| OSw x PHY x ES |  |  |  |  |  | -0.58 | 0.92 |
| RED x EFF x ES |  |  |  |  |  | -0.21 | 0.90 |
| OSw/o x EFF x ES |  |  |  |  |  | 0.92 | 0.86 |
| OSw x EFF x ES |  |  |  |  |  | -0.25 | 0.92 |
| RED x PER x ES |  |  |  |  |  | 0.63 | 0.90 |
| OSw/o x PER x ES |  |  |  |  |  | 0.40 | 0.86 |
| OSw x PER x ES |  |  |  |  |  | 0.75 | 0.92 |
| RED x FRU x ES |  |  |  |  |  | 1.05 | 0.90 |
| OSw/o x FRU x ES |  |  |  |  |  | 1.43. | 0.86 |
| OSw x FRU x ES |  |  |  |  |  | 0.36 | 0.92 |
| Task x Scale x OPE |  |  |  |  | F(15,7572.8)=0.89 |  |  |
| RED x TEM x OPE |  |  |  |  |  | 1.22 | 0.99 |
| OSw/o x TEM x OPE |  |  |  |  |  | -0.27 | 0.96 |
| OSw x TEM x OPE |  |  |  |  |  | 0.07 | 0.97 |
| RED x PHY x OPE |  |  |  |  |  | -0.44 | 0.99 |
| OSw/o x PHY x OPE |  |  |  |  |  | -0.78 | 0.96 |
| OSw x PHY x OPE |  |  |  |  |  | -0.08 | 0.97 |
| RED x EFF x OPE |  |  |  |  |  | 0.85 | 0.99 |
| OSw/o x EFF x OPE |  |  |  |  |  | -0.48 | 0.96 |
| OSw x EFF x OPE |  |  |  |  |  | 0.45 | 0.97 |
| RED x PER x OPE |  |  |  |  |  | 0.98 | 0.99 |
| OSw/o x PER x OPE |  |  |  |  |  | -0.27 | 0.96 |
| OSw x PER x OPE |  |  |  |  |  | 0.48 | 0.97 |
| RED x FRU x OPE |  |  |  |  |  | 1.39 | 0.99 |
| OSw/o x FRU x OPE |  |  |  |  |  | 0.52 | 0.96 |
| OSw x FRU x OPE |  |  |  |  |  | 1.78. | 0.97 |
|  |  |  |  |  |  |  |  |
| df | 87 |  |  |  |  | 157 |  |
| Δdf | 25 |  |  |  |  | 75 |  |
| AIC | 52374.13 |  |  |  |  | 52455.48 |  |
| BIC | 52945.45 |  |  |  |  | 53549.34 |  |
| LL | -26105.07 |  |  |  |  | -26070.74 |  |
| Deviance | 52210.13 |  |  |  |  | 52141.48 |  |
| ΔDeviance | 73.06*** |  |  |  |  | 68.65 |  |

Note: B = parameter estimate; SE = standard error of the parameter estimate; RED = Redman; OSw/o: Operational Service Without Intervention; OSw = Operational Service With Intervention; TEM = Temporal Demand; PHY = Physical Demand; EFF = Effort; PER = dissatisfaction with performance; FRU = Frustration; df = degrees of freedom; EXT = Extraversion; AGR = Agreeableness; CON = Conscientiousness; ES = Emotional Stability; OPE = Openness; Δdf = difference in degrees of freedom with respect to the previous model; ACI = Akaike Information Criterion; BIC = Schwarz's Bayesian Information Criterion; LL=Log-likelihood; ΔDeviance = difference in degrees of freedom with respect to the previous model.

* = *p* < .05; ** = *p* < .01; *** = *p* < .001.
